# Predicting genetic interactions, cell line dependencies and drug sensitivities with variational graph auto-encoder

**DOI:** 10.3389/fbinf.2022.1025783

**Published:** 2022-12-02

**Authors:** Asia Gervits, Roded Sharan

**Affiliations:** School of Computer Science, Tel Aviv University, Tel Aviv-Yafo, Israel

**Keywords:** deep learning, variational graph auto-encoder, genetic interaction, drug sensitivity, cell-line dependency

## Abstract

Large scale cancer genomics data provide crucial information about the disease and reveal points of intervention. However, systematic data have been collected in specific cell lines and their collection is laborious and costly. Hence, there is a need to develop computational models that can predict such data for any genomic context of interest. Here we develop novel models that build on variational graph auto-encoders and can integrate diverse types of data to provide high quality predictions of genetic interactions, cell line dependencies and drug sensitivities, outperforming previous methods. Our models, data and implementation are available at: https://github.com/aijag/drugGraphNet.

## 1 Introduction

Large scale cancer genomics data provide crucial information about the disease and reveal points of intervention. However, systematic data haven been collected in specific cell lines and their collection is laborious and costly. Hence, there is a need to develop computational models that can predict such data for any genomic context of interest.

Several large scale data sets that can be used for developing such models exist. The cancer dependency map project pinpoints potential drug targets in cancer lines whose knockdown leads to decreased cell fitness (DepMap, 2021). Systematic genetic interaction screens conducted in yeast and in human provide complementary information on potential cell-specific targets (Lee et al., 2018). The GDSC project performs systematic drug screens to identify sensitive cancer cell lines (Iorio et al., 2016).

There is a plethora of previous methods to predict these large scale data sets. Genetic interactions have been predicted based on gene ontology information (Yu et al., 2016; Ma et al., 2018), mutation and expression data (Lee et al., 2018), protein-protein interaction (PPI) data (Hao et al., 2021) and by specifically-designed deep learning models (Ma et al., 2018; Cai et al., 2020). Gene dependencies have been predicted based on expression information (Itzhacky and Sharan, 2021; Lin and Lichtarge, 2021), pathway information (Lin and Lichtarge, 2021), genetic essentiality profiles (Wang et al., 2019), and PPI and genomic alteration information (Benstead-Hume et al., 2019). Drug sensitivity data have been predicted based drug structure information combined with gene ontology information (Kuenzi et al., 2020) or gene expression data (Wang et al., 2017; Zhang et al., 2018; Choi et al., 2020; Itzhacky and Sharan, 2021). However, each method uses different information sources and most are geared toward a single prediction task.

Graph convolution networks (GCNs) and variational graph auto encoders (VGAEs) are powerful neural network architectures on graphs that can effectively capture the graph structure, perform node classifications and link prediction and are widely applicable (Scarselli et al., 2008; Kipf and Welling, 2016; Li et al., 2019). These techniques were also employed in the cancer genomics domain but again targeting a single task each time (Cai et al., 2020; Fan et al., 2020; Kuenzi et al., 2020; Ding et al., 2021; Hao et al., 2021).

In this paper, we develop an integrated model that combines VGAE with gene ontology information to perform a wide range of predictions spanning genetic interactions, gene dependencies and drug sensitivities. Our model is the first to propagate gene ontology information within a combined network of genetic interactions, gene-cell line relations and drug-target relations. It is shown to outperform previous methods for each of the prediction tasks. Its unique features include a new normalization layer and a modular architecture that allows the prediction of multiple attributes, represented as links in this model.

## 2 Methods

### 2.1 Data collection

#### 2.1.1 Genetic interaction (GI) data

We used genetic interactions from three different sources: (i) A yeast GI dataset from (Costanzo et al., 2016) downloaded from https://thecellmap.org/costanzo2016/. We used the provided thresholds of *p*-value threshold ≤0.05 and GI score ≤0.08, to extract ∼240K negative GIs. For the neutral pairs we used the same *p*-value threshold and score higher than 0.08. (ii) A human GI dataset collected in 2 cell lines, K562 and Jurkat, from (Horlbeck et al., 2018). We focused on the larger K562 dataset due to the high correlation between the two datasets. We used the reported threshold of -3, resulting in 1,678 negative GIs. (iii) SynLethDB collection of human synthetic lethality (SL) interactions from (Guo et al., 2016) with 19,667 SL pairs among 6,375 genes.

#### 2.1.2 Achilles gene dependency data

We downloaded gene dependency data from https://depmap.org (Dempster et al., 2019; DepMap, 2021), version 21Q4. We used a dependency threshold of 0.5 as in the recently published method (BioVNN, described below) (Lin and Lichtarge, 2021). For constructing our model we also downloaded CCLE (Ghandi et al., 2019) mutations for each cell line and selected the damaging mutations by the variant annotations. We excluded genes which were either nearly all dependent (up to 6) across cell lines, as in BioVNN. The final constructed dataset contains 922 cell lines and their affect among 5,975 genes, spanning ∼1.4M dependent pairs.

#### 2.1.3 Drug sensitivity data

We downloaded the GDSC binarized IC50 dataset from http://www.cancerrxgene.org/(Iorio et al., 2016). The dataset consists of 1,001 cancer cell line and 265 tested drugs, spanning ∼20K sensitive pairs. In addition, we downloaded from the same site the gene targets for each drug and the mutated genes for each cell line that were used to construct our model. Due to lack of variant annotation information in this datasource, we focused on nonsense, frame shift, exonic splicing silencer and gene fusion mutations that we considered as harmful.

#### 2.1.4 Gene ontology (GO)

For our feature generation we downloaded the latest version of ontology file from http://geneontology.org/. We used all the terms from the three GO subnetworks: biology process (BP), cellular components (CC) and molecular functions (MF). Following the original publication we removed terms with the evidence code ‘inferred by genetic interaction’ (IGI), to avoid potential circularity in predicting genetic interactions. In addition, terms that do not connect to any gene in the model’s graph were removed.

### 2.2 Link prediction algorithm

Graph autoencoder (GAE) and variational graph autoencoder (VGAE) models have been demonstrated as efficient tools to learn graph embeddings in an unsupervised way and serving as an infrastructure for link prediction (Kipf and Welling, 2016). Here we combined the VGAE model with gene ontology information to generate a modular graph structure that models the connections between drugs, cell lines and genes and could be used to a wide range of tasks: predicting genetic interactions, cell line dependencies and drug sensitivities.

Given a graph *G* on a set of *n* vertices *V* and a real adjacency matrix *A*, a graph convolutional network (GCN) model receives two matrices as inputs: *A* ∈ *R* ^*n*×*n*^ and *X* ∈ *R* ^*n*×*f*^ as the feature matrix of *V*. The output of a single layer is $\sigma \left(\hat{A}\delta \left(X\right)W\right)$ Where *σ* is the activation function, *δ* is a dropout applied on the input, $\bar{A}≔A+I$ , $\bar{D}$ the corresponding diagonal degree matrix and $\hat{A}≔{\bar{D}}^{-\frac{1}{2}}\bar{A}{\bar{D}}^{-\frac{1}{2}}$ , *W* the learnable weight matrices.

#### 2.2.1 Encoder

In our model we use a normalization layer that we find to improve model performance, followed by a 2-layer GCN which computes the node embedding distribution by Eqs. 1, 2: *μ* ∈ *R* ^*n*×*f*^ is the matrix of mean vectors, log  *σ* ^2^ ∈ *R* ^*n*×*f*^ is (log of) the variance matrix, and *f* represents the dimension of the embedding node vectors. From those distribution matrices we draw the embedding matrix *Z* by *z* = *μ* + *σ* ∗ *ϵ*, where ϵ∼N(0,1) . In the equations, *σ*_*j*_ are the activation functions, *W*_*i*_s are learnable weight matrices and *b* is a learnable bias vector. The contribution of the additional normalization layer, compared to the standard VGAE performance, is summarized in Supplementary Figure S1.

$$\bar{X}=XW_{0}+b$$ (1)

μ=σ2A^σ1A^X¯W1W2;log σ2=σ2A^σ1A^X¯W1W3 (2)

#### 2.2.2 Decoder

The decoder is defined by the dot product between latent *Z* variables, and the output is a reconstructed adjacency matrix $\tilde{A}$ as follows:

$$\tilde{A}=\sigma_{3}\left(ZZ^{T}\right)$$ (3)

where *σ*_3_ is the sigmoid function.

#### 2.2.3 Loss function

The loss function of VGAE includes two parts. The first part is the binary cross-entropy between the target *A* and the model output, while the second part is the KL-divergence between $q\left(Z|X,A\right)=\Pi_{i=1}^{N}q\left(z_{i}|X,A\right)=\Pi_{i=1}^{N}N\left(z_{i}|\mu_{i},\mathrm{diag}\left(\sigma_{i}^{2}\right)\right)$ and $p\left(Z\right)=\Pi_{i}p\left(z_{i}\right)=\Pi_{i}N\left(z_{i}|0,I\right)$ , this part aims to generate the latent dimension with Gaussian distribution. The final loss function is defined as follows:

$$L=E_{q\left(Z|X,A\right)}\left[\log p\left(A|Z\right)\right]-KL\left[q\left(Z|X,A\right)\|p\left(Z\right)\right]$$ (4)

**FIGURE 1:**
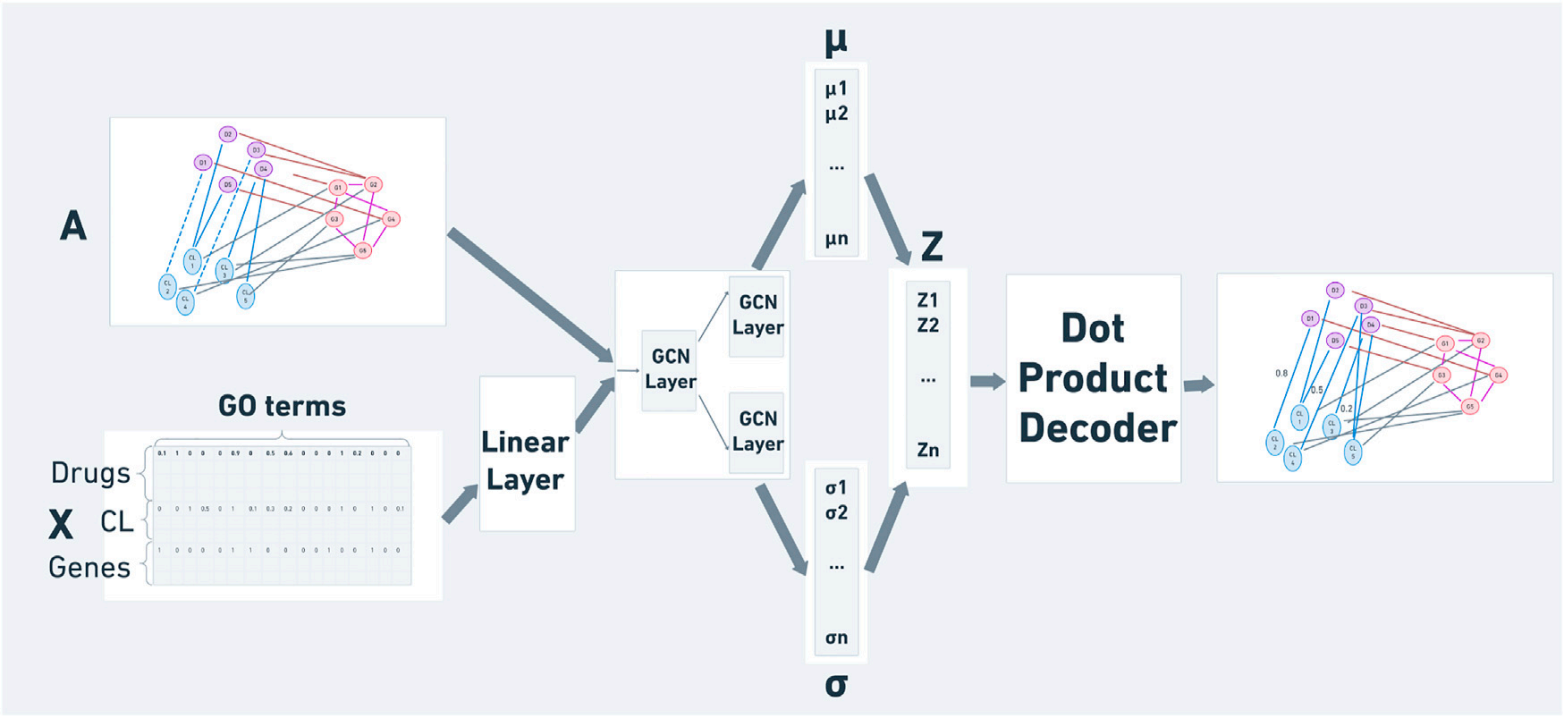
An illustration of our final constructed model for drug sensitivity prediction. The adjacency matrix *A* with the masked sensitivity interactions and feature matrix *X* serve as inputs. The final output is the reconstructed $\tilde{A}$ adjacency matrix, allowing the prediction of new drug-cell line interactions.

The full model we developed is illustrated in Figure 1. Details about the underlying graphs and features are provided below.

#### 2.2.4 Graph construction

**FIGURE 2:**
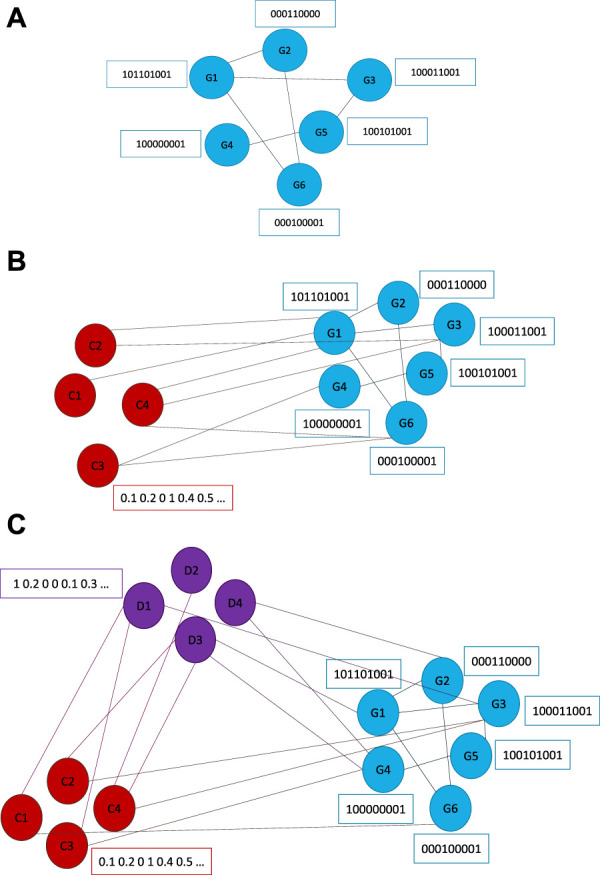
Graph model structures that underlie our VGAE model. **(A)** *VGAE*_*G*_—GI part with gene nodes. **(B)** *VGAE*_*CD*_—Cell line dependency part with nodes representing genes and cell lines. **(C)** *VGAE*_*DS*_—Drug sensitivity part with nodes representing genes, cell-lines and drugs.

The graphs underlying our models are gradually built in three parts. The first part is a graph of GIs only (Figure 2A), here the nodes represent genes and the edges represent GIs. We will represent this model as *VGAE*_*G*_. The second part are nodes representing cell lines that are connected to the first part nodes using dependency relations (Figure 2B), we will represent this model as *VGAE*_*CD*_. The final part consists also of drug nodes which are connected to the gene part using drug-target relations. The connections between drugs and cell lines represent the drug sensitivity, which is the target of our prediction in this model, we will represent this model as *VGAE*_*DS*_ (Figure 2C). In the *VGAE*_*DS*_ graph, cell lines are connected to their mutated genes (rather than using dependency relations like in *VGAE*_*CD*_).

#### 2.2.5 Feature generation

To generate the features vector for each node we used the ontotype method (Yu et al., 2016), where each gene is represented by a binary vector of its GO terms and a gene set by the sum of its member gene vectors. Specifically, we associated cell-lines nodes with their sets of mutated genes, and drug nodes with their sets of gene targets. All nodes vectors were normalized by dividing by the number of genes they represent. The final features included the terms that were connected to at least one of the drugs, cell lines or genes, so all the three types of nodes is sharing the same features dimension. GO annotations with the evidence code “inferred by genetic interaction” (IGI) were removed to avoid potential circularity in predicting genetic interactions.

### 2.3 Training procedure and performance evaluation

To evaluate our model we performed five-fold cross-validation (CV). For highly imbalanced datasets, like drug sensitivity, we also generated temporary balanced datasets by randomly sampling neutral samples of the same size as the positive samples, and performed 10 repetitions of the CV. For our model training we used randomly selected 10% edges from the training data as a validation set. The validation set was used for the evaluation of the model during the training for selecting the model from the epoch best performances, and for early stopping of the training process in the case of overfitting. We optimized the model’s hyperparameters using the validation data, choosing the hyperparameter configuration that performed best.

As a benchmark in all prediction tasks we compared to the ontotype method (Yu et al., 2016) that we build on. In this method, the ontotype feature vectors are fed into a random forest (RF) classifier in the prediction phase rather than being propagated in a graph as in our new model. For the RF training, we used the same split on the edges. The input features for the cell dependency and drug sensitivity tasks are the sum of the cell line and drug features that we used in our model.

We calculated true positive rate (TPR), false positive rate (FPR), precision and recall by varying the preset thresholds to construct receiver operating characteristic (ROC) and precision–recall (PR) curves. We then generated two metrics, namely the area under the ROC curve (AUROC) and the area under the PR curve (AUPRC), to evaluate the performance of our model and other methods. We averaged the results from all cross-validation splits to calculate the overall AUROC and AUPRC.

## 3 Results

### 3.1 *VGAE*_*G*_ model for predicting genetic interactions

**FIGURE 3:**
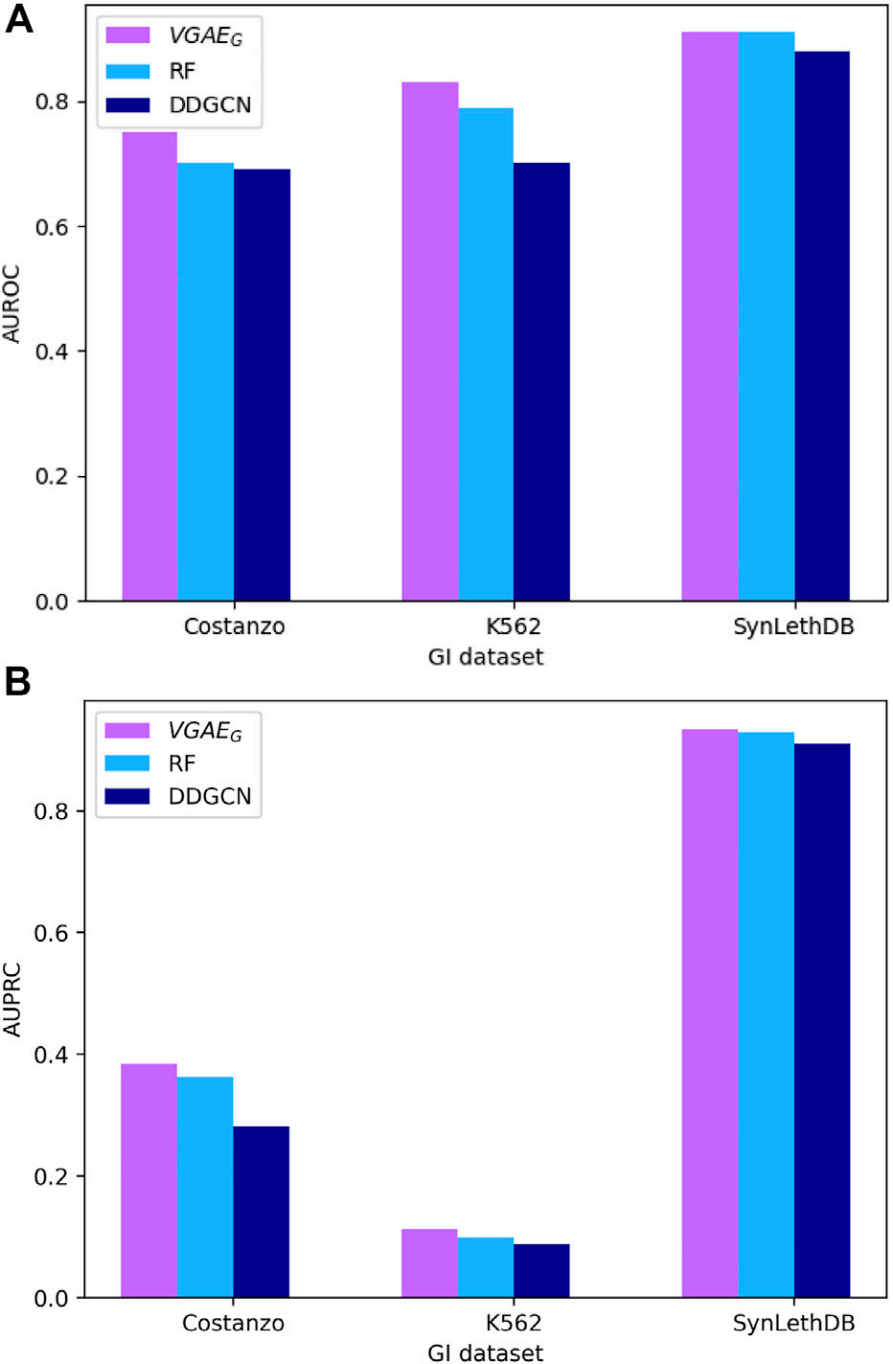
Performance of GI prediction across different datasets. **(A)** AUROC results. **(B)** AUPRC results.

To evaluate our VGAE model we applied its first variant *VGAE*_*G*_ to predict genetic interactions and compared to the ontotype method (Yu et al., 2016). We tested three data sets from yeast (systematic GI data) and human (systematic data from the K562 cell-line and interactions from SynLethDB) as described in the Data section of the Methods. Our model outperformed the previous method (Figure 3; Supplementary Table S1). To analyze the contribution of our model on top of the ontotype method, we split the systematic datasets to two equal parts based on the ontotype sparsities, observing that the higher the sparsity the higher the contribution (Supplementary Table S2). In addition we also compared our model to a recent GCN model, DDGCN (Cai et al., 2020), that does not use the GO knowledge or the additional normalization layer. The results show the benefit of integrating GO information within a GCN and motivate the more complex models below.

### 3.2 *VGAE*_*CD*_ model for predicting cancer dependencies

**FIGURE 4:**
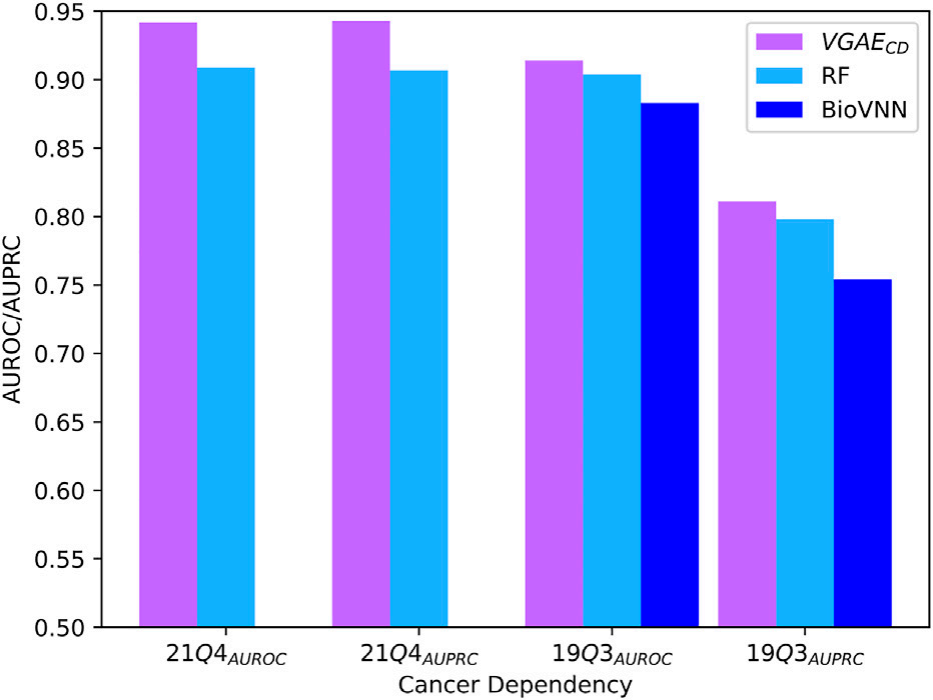
Performance evaluation for cancer dependency prediction on the two evaluated datasets. The presented BioVNN results are taken from (Lin and Lichtarge, 2021).

Next, we tested our second model variant *VGAE*_*CD*_ on the recent 21Q4 cell-line dependency dataset. In this application the input genetic interactions were taken from SynLethDB. For comparison purpose, we adapted the ontotype method to this setting, representing a cell line by the normalized sum of ontotype vectors of genes it depends on. In addition, we compared our model to a recently published method (BioVNN) which uses a visible neural network over pathway knowledge to predict dependencies (Lin and Lichtarge, 2021). For this comparison, we analyzed the same 19Q3 dataset used in the previous paper containing 609 cell-lines and 683 genes. We also employed the same cross validation procedure where cell lines are distinct between the train, validation and test folds. The AUROC and AUPRC results are summarized in Figure 4; Supplementary Table S1, and again show the superiority of our model.

### 3.3 *VGAE*_*DS*_ model for predicting drug sensitivity data

**FIGURE 5:**
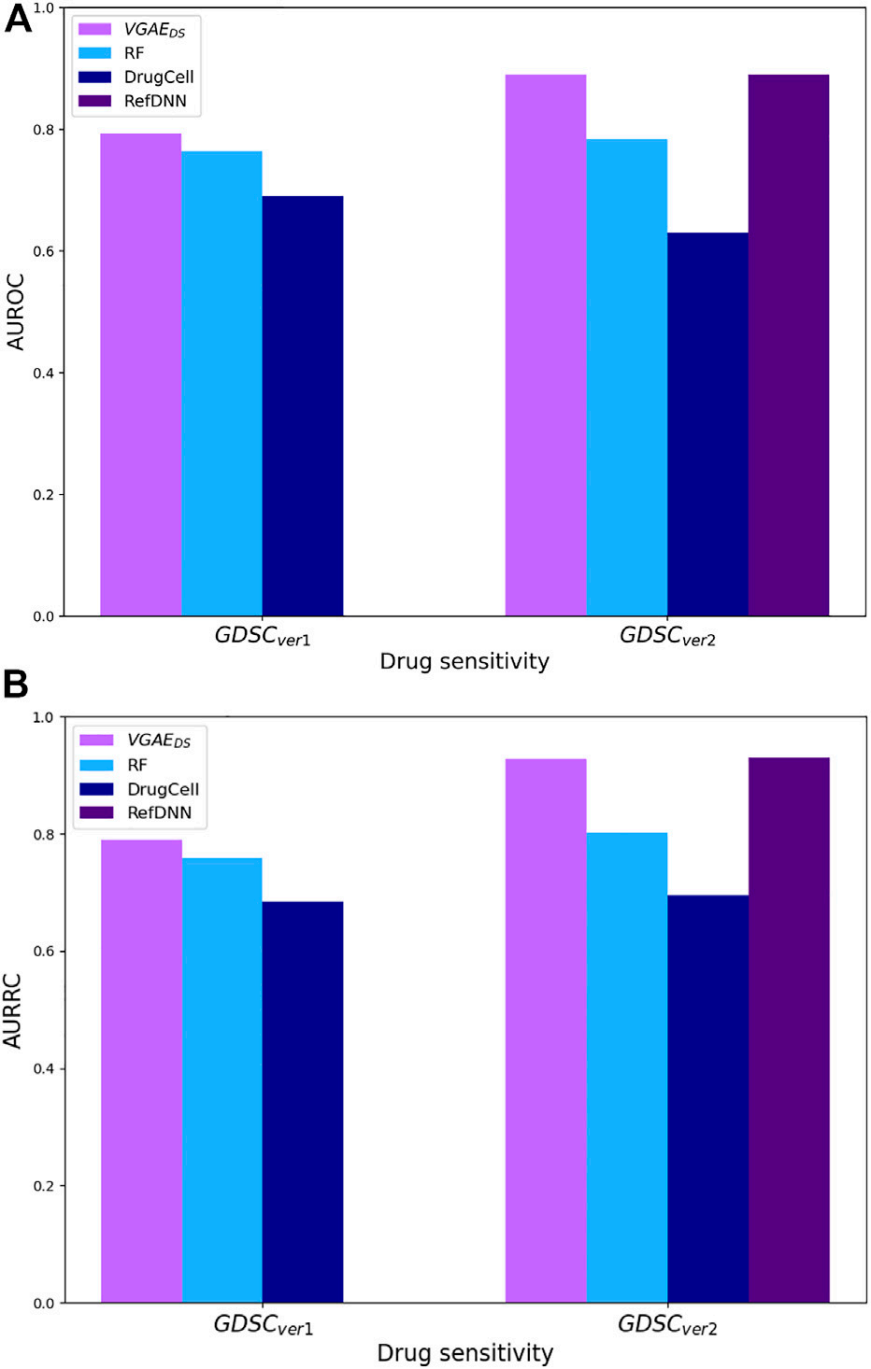
Prediction performance on drug sensitivity data. **(A)** AUROC results. **(B)** AUPRC results.

Last, we applied our full model to predict drug sensitivity relations. For comparison purpose we again adapted the ontotype method for this task by representing each drug (cell line, resp.) by the normalized sum of the ontotype vectors of its targets (mutated genes, resp.). We further compared ourselves to DrugCell (Kuenzi et al., 2020), a deep network that similarly to our model uses GO information and cell-line mutations, but unlike our model uses drug chemical structure as additional input. Since DrugCell is designed for a regression task, we adapted it for classification by changing: (i) the last activation function to sigmoid activation; and (ii) the loss function to binary cross-entropy. In addition, we tested our model on the different version of GDSC dataset (version 17.3) that was used for the training of a recently published method (RefDNN), a deep NN that uses gene expression and drug structure as inputs (Choi et al., 2020). In this dataset, IC50 continuous values were binarized based on the reported maximum screening concentration threshold. The comparison results are summarized in Figure 5; Supplementary Table S1, and show that *VGAE*_*DS*_ outperforms the other methods or receives similar results in both datasets.

## 4 Discussion

We have presented a graph variational auto-encoder based model for predicting genetic interactions, cell line dependencies and drug sensitivities. The model propagates gene ontology information over a network of gene, drug and cell-line interactions, providing uniform representations to genes, cell lines and drugs, allowing the wide scale of predictions. The unique features of the model include a new normalization layer and a modular architecture that allows the prediction of multiple attributes. While our models achieved promising results, their performance in a real clinical setting where samples come from real patients will need to be assessed when such data becomes available.

For future work, we would like to create one model that can predict genetic interactions, cell line dependencies and drug sensitivities, rather than having three separate models. To this end, the connections between cell lines and genes would represent cancer dependency instead of cell lines mutations. This model structure is currently not feasible due to the low number of overlapping cell lines between dependency and sensitivity data.

## Data Availability

The datasets presented in this study can be found in online repositories. The names of the repository/repositories and accession number(s) can be found in the article/Supplementary Material.
