# Supplementary material for: Predicting genetic interactions, cell line dependencies and drug sensitivities with variational graph auto-encoder
Source: Front Bioinform. 2022 Dec 2;2:1025783. doi: 10.3389/fbinf.2022.1025783 (PMC9755598; doi:10.3389/fbinf.2022.1025783)
Supplement: Supplementary file 1 [file DataSheet1.PDF]

Supplementary Material

1 Supplementary Figures and Tables

1.1 Supplementary Figures

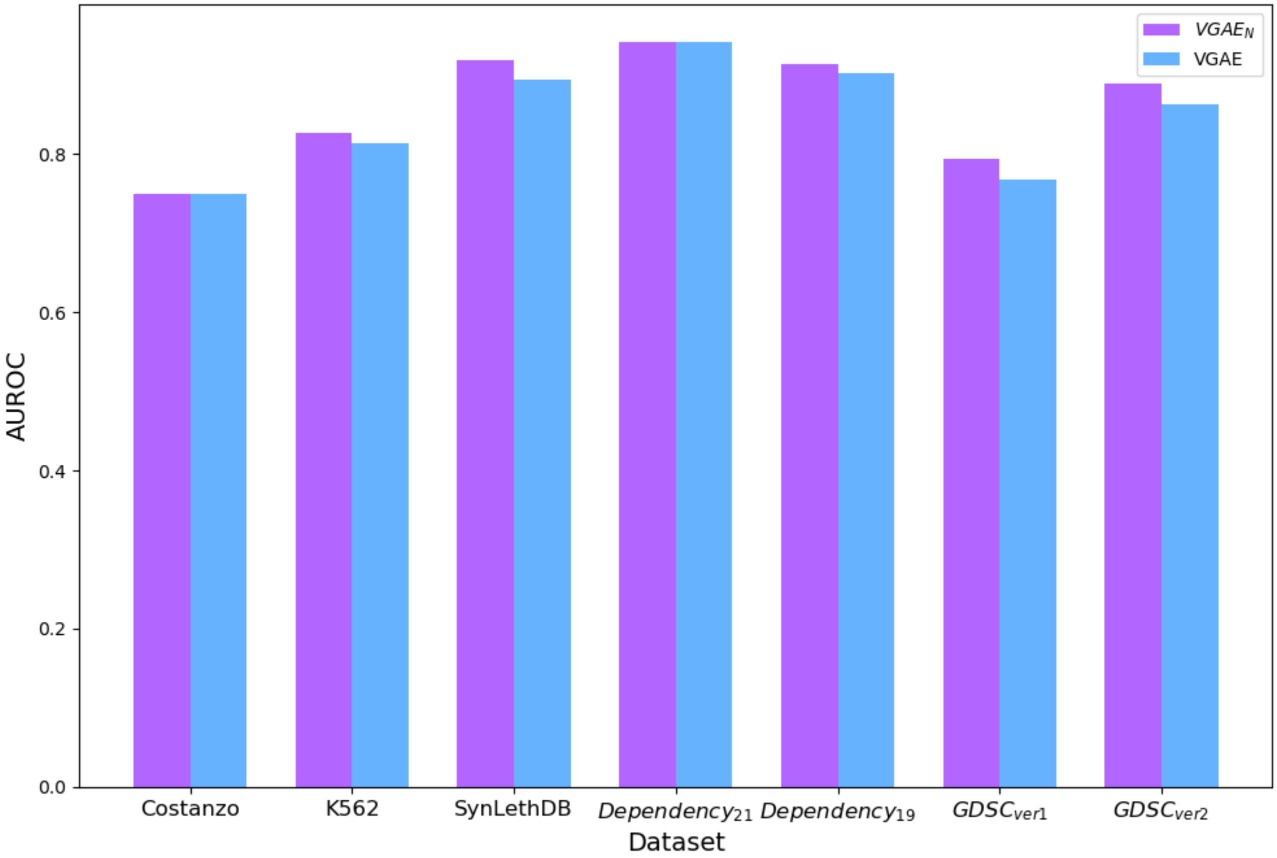

Supplementary Figure 1. This graph demonstrates the contribution of the additional normalization layer compared to the standard VGAE performance. Here VGAEN represent the performance of our model compared to the results of the same model without the normalization layer. The performance of this model improved in up to 0.03 AUROC scores, without reducing the performance in all the datasets and prediction targets.

1.2 Supplementary Tables

		AUROC/AUPRC			
		**$VGAE_G$**	**RF**	**DDGCN**	
GI	Costanzo	0.750/0.384	0.704/0.363	0.686/0.303	
	K562	0.826/0.113	0.787/0.099	0.693/0.085	
	SynLethDB	0.918/0.934	0.912/0.929	0.878/0.892	
		**$VGAE_{CD}$**	**RF**	**BioVNN**	
Cancer Dependencies	$Dependency_{21}$	0.942/0.943	0.909/0.907	-	
	$Dependency_{19}$	0.914/0.811	0.904/0.798	0.883/0.754	
		**$VGAE_{DS}$**	**RF**	**DrugCell**	**RefDNN**
Drug Sensitivity	$GDSC_{ver1}$	0.793/0.794	0.764/0.758	0.687/0.684	-
	$GDSC_{ver2}$	0.889/0.933	0.785/0.799	0.641/0.675	0.891/0.932

Supplementary Table 1. This table contains all the numeric results from all our experiments, all the results are the average of 5 CV folds with $p < 0.01$.

Dataset	Sparsity	VGAE- AUROC	RF - AUROC	Improvement
K562	High	0.817	0.769	0.048
	Low	0.835	0.805	0.031
Costanzo	High	0.746	0.679	0.067
	Low	0.754	0.721	0.033

Supplementary Table 2. This table demonstrates the benefit of our model in the classification of samples with sparse ontotype representations, compared to the previous method of RF when predicting genetic interactions. The presented AUROC results are the average of 5 CV folds, each iteration was split to two equal datasets with high and low sparsity.
